# Behaviour, barriers and facilitators of shared decision making in breast cancer surgical treatment: A qualitative systematic review using a ‘Best Fit’ framework approach

**DOI:** 10.1111/hex.14019

**Published:** 2024-04-01

**Authors:** Hongying Zheng, Linning Yang, Jiale Hu, Yan Yang

**Affiliations:** ^1^ School of Nursing, School of Medicine Shanghai Jiao Tong University Shanghai China; ^2^ Department of Nurse Anesthesia, College of Health Professions Virginia Commonwealth University Richmond Virginia USA; ^3^ Department of Nursing, Renji Hospital Shanghai Jiao Tong University School of Medicine Shanghai China

**Keywords:** barriers, breast cancer, facilitators, shared decision making, surgical decision‐making behaviour

## Abstract

**Background:**

Due to the diversity and high sensitivity of the treatment, there were difficulties and uncertainties in the breast cancer surgical decision‐making process. We aimed to describe the patient's decision‐making behaviour and shared decision‐making (SDM)‐related barriers and facilitators in breast cancer surgical treatment.

**Methods:**

We searched eight databases for qualitative studies and mixed‐method studies about breast cancer patients' surgical decision‐making process from inception to March 2021. The quality of the studies was critically appraised by two researchers independently. We used a ‘best fit framework approach’ to analyze and synthesize the evidence.

**Results:**

Twenty‐eight qualitative studies and three mixed‐method studies were included in this study. Four themes and 10 subthemes were extracted: (a) struggling with various considerations, (b) actual decision‐making behaviours, (c) SDM not routinely implemented and (d) multiple facilitators and barriers to SDM.

**Conclusions:**

Patients had various considerations of breast surgery and SDM was not routinely implemented. There was a discrepancy between information exchange behaviours, value clarification, decision support utilization and SDM due to cognitive and behavioural biases. When individuals made surgical decisions, their behaviours were affected by individual‐level and system‐level factors. Therefore, healthcare providers and other stakeholders should constantly improve communication skills and collaboration, and emphasize the importance of decision support, so as to embed SDM into routine practice.

**Patient and Public Contribution:**

This systematic review was conducted as part of a wider research entitled: Breast cancer patients' actual participation roles in surgical decision making: a mixed method research. The results of this project helped us to better analyze and generalize patients' views.

## 1 INTRODUCTION

In recent years, the prevalence and mortality of breast cancer have been increasing.^1^ Global Cancer Statistics 2018 estimated that breast cancer accounted for 24.2% of new cancer cases and 15.0% of cancer deaths in women, both ranking first.^1^ Breast surgery is the criterion standard of treatment for breast cancer. Common breast cancer surgeries include a mastectomy, modified radical mastectomy (MRM), breast‐conserving therapy (BCT), unilateral mastectomy (UM), contralateral prophylactic mastectomy (CPM) and breast reconstruction (BR). The trade‐off^2^, ^3^ between ‘breast preservation’ or ‘breast removal’ and ‘reduction of cancer recurrence’ or ‘quality of life’, brought more challenges to patients and their families. Because the breast is a vital organ related to body image and self‐esteem for women. When women lose a breast, they may experience psychosocial dysfunction, such as anxiety, stress and an impaired quality of life after treatment.^4^ For reconstructive surgery, most patients were hesitant between body image, cosmetic results, family finances and the pain of a second operation. Due to the diversity and high sensitivity of the treatment of breast cancer patients, there were difficulties and uncertainties^3^, ^5^ in the decision‐making process. Therefore, it is important to understand the patient's wishes and encourage active participation in the decision making process for breast cancer surgery to minimize these negative outcomes.

For decades, the concept of patient participation in medical decision making has attracted wide attention in the healthcare field. Shared decision making (SDM) has been well recognized as an essential element of patient‐centred care. Applying SDM could improve patients' knowledge, reduce patients' suffering, actively elicitation of preferences and values and promote patients' involvement and quality of surgical care practice.^6^, ^7^ Concerns about the appropriateness of SDM may be particularly pronounced in surgical decision making given the often dramatic and irreversible outcomes associated with surgery.^8^ However, the use of SDM in surgery was still in its infancy,^8^, ^9^, ^10^ despite positive feedback from both clinicians and patients.^11^

The implementation of SDM was influenced by multiple and complex factors,^5^, ^9^, ^10^, ^12^, ^13^ such as patient factors, surgeon factors, healthcare organization factors and external environmental factors, especially cultures and local practice patterns. At the same time, the existing studies lack systematic and intrinsic logical correlation of influencing factors, which indicates the need for further analysis based on theoretical frameworks. To our knowledge, some reviews^4^, ^5^, ^13^ summarizing the experiences of patients undergoing breast cancer surgery are now available, but there is a lack of comprehensive understanding of the barriers and facilitators associated with SDM, which reveals the lack of attention of most researchers to this important topic. This knowledge gap is a barrier to the provision of effective decision support and patient‐centred care by healthcare providers (HCPs).

To fill this gap, this study conducted an integrative review to integrate the relevant qualitative evidence from qualitative studies and mixed‐method studies. This study aimed to answer the following questions in breast cancer surgical treatment: (a) What is the patient's decision‐making behaviour? (b) What evidence exists regarding the facilitators and barriers related to SDM in clinical practice?

## 2 METHODS

This study combined the qualitative systematic review approach with the ‘best fit’ framework synthesis (BFFS), which provided a practical and rapid method for qualitative evidence synthesis. This study followed the Preferred Reporting Items for Systematic Reviews and Meta‐Analyses statement.^14^ We have written the research protocol for this systematic review, but not registered or published.

### 2.1 Literature search

We searched Cochrane Library, Pubmed, Embase, CINHAL, PsycINFO, Chinese Biology Medicine disc, Chinese National Knowledge Infrastructure and Wangfang database for qualitative studies and mixed‐method studies about breast cancer patients' surgical decision making from inception to March 2021. Further searching and supplementing were carried out through the references. Details of the search strategies can be found in Supporting Information S1: Appendix A.

### 2.2 Inclusion and exclusion criteria

Inclusion criteria were (a) *population*: breast cancer patients; (b) *interest of phenomenon*: surgical decision‐making behaviours and SDM behaviours; (c) *context*: surgical decision including mastectomy, BCT, MRM, UM, CPM and BR and (d) *study design*: the qualitative study (grounded theory study, phenomenological study, action study and narrative study) and mixed‐method study. Exclusion criteria were (a) duplicate publication; (b) unavailable to get the full text; (c) conference abstract and (d) research protocol.

### 2.3 Literature screening

Two researchers (H. Z. and L. Y.) searched the literature independently, and the retrieved literature records were imported into NoteExpress software to remove duplicates. Two researchers independently screened the title and abstract. Articles with unrelated study design, research objectives, participants and context were eliminated. And then, two researchers read the full‐text articles to eliminate the inconsistent studies. Any discrepancy was settled by discussion and resolved by a third researcher (Y. Y.).

### 2.4 Data extraction

This systematic review extracted qualitative data from qualitative and mixed studies. Two researchers (H. Z. and L. Y.) independently extracted relevant information from the articles, including population, the interest of phenomenon, context, study design, and so forth. After data extraction, two researchers cross‐checked the extraction results. The data were examined by the third researcher (J. H.) qualified in the health and social sciences to obtain data validation.

### 2.5 Quality evaluation

The quality of studies was critically appraised by two researchers (H. Z. and L. Y.) independently using the Mixed Methods Appraisal Tool (MMAT) version 2018.^15^ MMAT was developed for critically appraising different study designs, including qualitative, quantitative and mixed‐method studies. MMAT consists of 5 subscales and 27 items. The first subscale (including entries 1.1–1.5) of MMAT was used to evaluate qualitative studies, and the fifth subscale (Including entries 5.1–5.5) was used to evaluate mixed‐method studies. It was advised to provide a more detailed presentation of the ratings of each criterion rather than calculating an overall score. In this study, the number of items with the option of ‘yes’ in the quality evaluation items was counted. ‘0’ means none of the items were satisfied, and ‘5’ means all of the items were satisfied. In this study, studies with poor study quality will be excluded to reduce the bias of the study results. Any dispute shall be settled by discussion and arbitration by the third researcher (Y. Y.).

### 2.6 Data synthesis

#### 2.6.1 Framework selection

We synthesized the qualitative data based on Andersen's Behavioral Model of Health Services Use^16^ and the interprofessional SDM (IP‐SDM) model.^17^ Andersen's model is widely used to study health service utilization. Clinical SDM is a component of broader health service utilization. The model identifies factors that enable or hinder an individual's access to healthcare services. Key variables in the model include contextual characteristics, individual characteristics, health behaviours and outcomes. This study utilized Andersen's model as a guiding framework to analyze the logical links between influencing factors, decision‐making behaviours and decision outcomes. Additionally, due to the specificity of clinical SDM, the IP‐SDM model was also employed. The IP‐SDM model refines the specific steps of SDM, including (a) patient with a health condition, (b) information exchange, (c) value clarification, (d) feasibility, (e) actual decision making and (f) implementation. This study employed the IP‐SDM model to refine these specific behaviours.

#### 2.6.2 BFFS

We followed the BFFS^18^ method, proposed by Booth and Carroll. It allowed for both a deductive analysis using an a priori framework and an inductive analysis based on new themes from selected studies that are not part of the a priori framework. The approach therefore was augmentative and deductive (building on this existing model or framework), rather than grounded or inductive (starting with a completely blank sheet). The model identified did not entirely match the topic under study, but it was a ‘best fit’ and provided a relevant pre‐existing framework and themes against which to code the data from the studies identified for this review. Andersen's Model and IP‐SDM model provided the a priori framework of themes against which to open code the data extracted from the included studies. The preliminary conceptual framework was extended to generate the final result. Priori framework of themes based on Anderson's Model and IP‐SDM model can be found in Table 1.

**Table 1:** Priori framework of themes and subthemes.

Theme	Subthemes
Barriers and facilitators	Individual characteristics Contextual characteristics
Decision behaviours	Step 1 patient with a health condition Step 2 exchange of information Step 3 values clarification Step 4 feasibility of the options Step 5 actual decision Step 6 implementation
Decision outcome	Shared decision making Routine decision making

The results of the literature were integrated using the Nvivo software version 11. Based on understanding the methodology and philosophy of each germplasm research, researchers (H. Z. and L. Y.) constantly read to be familiar with the data, identified the meaningful units of relevant data, open‐coded, developed categories and themes and iterative data. Relevant excerpts were coded as new or existing nodes and tree nodes were created from these nodes. Coding to these nodes continued with the rest of the literature. At the same time, node and tree nodes were created and revised constantly during this process.

## 3 RESULTS

### 3.1 Study selection

**Figure 1:**
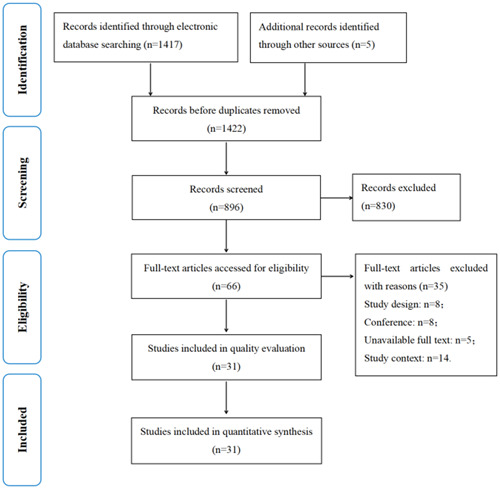
Preferred Reporting Items for Systematic Reviews and Meta‐Analyses flow diagram of the study screening process.

A total of 1422 records were retrieved, and these records were imported into the NoteExpress reference management software. Then 526 duplicates were removed. After preliminary screening of the title and abstract and reading the full text, 31 articles were finally selected (Figure 1).

#### 3.1.1 Study characteristics

Twenty‐eight qualitative studies and three mixed‐method studies were included in this study. These included studies were from the United States, the United Kingdom, China, Canada and other countries. The surgical decision‐making situation included a mastectomy, BCT, UM, CPM and BR decision (Table 2).

**Table 2:** Characteristics of the included studies.

ID	Reference	Country		Study design	Population	Interest of phenomena	Context	Brief finding
*Qualitative study*								
1	Lally^19^		United States	Semistructured interview	12 Breast cancer patients	Pretreatment thoughts and behaviours	Surgical decision	3 Themes: information processing; contemplating options (choosing a surgical treatment, motivations for choice, decision‐making ease); interacting with others (HCPs, family, friends, and others)
2	Begum et al.^20^		United Kingdom	Semistructured interview	21 Patients underwent BR	Experiences of the decision to undergo BR surgery following mastectomy	BR decision	4 Themes: reasons for BR, reasons for immediate reconstruction, reasons for delayed reconstruction, provision of information regarding BR
3	Caldon et al.^21^		United Kingdom	Semistructured interview	65 Breast cancer patients	Relationship between mastectomy rate and decision‐making experience	Mastectomy or BCT decision	2 Themes: patient‐specific themes (most reassuring treatment option, least disruptive treatment option); breast unit‐specific themes (information content and style, time and process of decision making, autonomy, level of patient participation in decision making)
4	Fang et al.^22^		China	Descriptive qualitative design	10 Taiwanese patients underwent mastectomy	Experience about facing a mastectomy	Surgical decision	4 Themes: surviving is a priority, fulfilling responsibility, coming to terms with postsurgery alternatives, making arrangements
5	Obeidat et al.^23^		United States	Heideggerian hermeneutical methodology	10 Arab American women with ESBC	Experience of undergoing surgical treatment	Surgical decision	5 Themes: breast cancer as a life‐threatening illness, breast cancer as a fate, seeking information about own diagnosis and treatment, trusting physicians for treatment choice and/or decisions, accessing/finding social support
6	Swainston et al.^24^		United Kingdom	Hermeneutic phenomenological method	20 Breast cancer patients	Lived experience and the treatment decision‐making process over time	Surgical decision	4 Themes: role in the treatment decision‐making process, acceptance of medical opinions, getting rid of it, cancer schemas
7	Abdullah et al.^25^		Malaysia	Qualitative study	8 Malaysian ESBC patients	Decision‐making experience	Surgical decision	4 Themes: discovery (prediagnosis), confirmatory (receiving bad news), deliberation, decision (making a decision)
8	Obeidat et al.^26^		Jordan	Semistructured interview	28 ESBC patients underwent surgery	Health‐related information exchange experience	Surgical decision	3 Themes: knowledge about breast cancer and its treatment, communication of cancer diagnosis and treatment, educating on treatment side effects
9	Covelli et al.^27^		Canada	Grounded theory	29 ESBC patients	treatment choice and influencing factors for more extensive surgery	UM or CPM decision	3 Themes: decision‐making experience, reasons for mastectomy, postoperative outcomes
10	Potter et al.^28^		United Kingdom	Semistructured interview	31 Patients considering BR and 35 HCPs	Adequacy of information provided for decision‐making in RBS	BR decision	3 Themes: information from health professionals, information from additional sources, patients’ perceptions of the adequacy of information for decision making
11	Ristevski et al.^29^		Australia	Semistructured interview	70 ESBC patients	Factors rural women perceived to influence the surgical choice	Surgical decision	2 Themes: patient‐led psychosocial factors, surgeon‐led factors
12	Schubart et al.^30^		United States	Interpretive description method	14 African‐Americans with breast cancer	Key issues regarding treatment decisions	Surgical decision	3 Themes: fear and worry, information sources and knowledge about breast cancer, support systems
13	Robinson et al.^31^		United States	Qualitative, videotape‐based analysis	132 Breast cancer patients	Information needs and information seeking	Surgical decision	15 Theme: asks for description of surgical procedure of lumpectomy/mastectomy, asks about type of surgical procedure needed or more/less appropriate/recommended, asks about date/timeframe when surgery could occur, asks for description of procedures involving testing/removing lymph nodes, asks about surgical recovery process/timeframe, asks about spread of cancer to lymph nodes/body, asks for description of procedure involving radiation therapy, asks about severity of cancer/tumour and/or its characteristics, asks about need/rationale for chemotherapy, asks about type of tumour involved, asks about need/rationale for radiation, asks for description of procedure involving reconstruction, asks about hormone therapy and its rationale, asks about hormone‐receptor and her2 status, asks about recurrence and its likelihood/detection
14	Brown et al.^32^		United Kingdom	Semistructured interview	34 Patients listed for RRM surgery	RRM decisions process	RRM decisions	5 Themes: RRM was generally a ‘no‐brainer’; decision making was dominated by fear and vulnerability; patients felt RRM to be applicable to them; doing ‘all I can’ to feel safe; where ‘deliberation' did not occur before the decision, it often occurred after
15	Fasse et al.^33^		France	Qualitative study	9 Breast cancer patients	Decision‐making process for BR after mastectomy	BR decision	2 Themes: external influence in the decision‐making process (personalized care, care provision, constraint experience, intimate decision); implication of the partner in the decision‐making process (common experience of care process, emotional help and reassurance, practical support, intimacy, tensions, consultative function)
16	Fu et al.^34^		United States	Qualitative study	35 Asian immigrant breast cancer patients	Cultural factors, values and perceptions that impact BR	BR decision	8 Themes: functionality, age, perceptions of plastic surgery, inconvenience, community and family, fear of implants, language, information
17	Gu et al.^35^		Canada	Interpretive description method	25 ESBC patients	Factors that influence the choice between BCT and mastectomy	mastectomy or BCT decision	2 Themes: choose mastectomy (worry about cancer recurrence, perceived consequences of BCT treatment, breast‐tumour size perception); choose BCT (mastectomy being too radical, surgeon influence, feminine identity)
18	Hasak et al.^36^		United States	Semistructured interview	20 Postmastectomy patients, 10 PMBR surgeons, 10 PMBR nurses	PMBR experience	PMBR decision	4 Themes: engagement in SDM is variable; stakeholders described many barriers to SDM, including limited information‐sharing, clinician pressure, and clinician biases; SDM was particularly challenging when patients and clinicians disagreed about the best PMBR option for a patient. however, those who engaged in SDM during disagreements often ended up with more satisfied patients; stakeholders described factors that facilitated SDM, including patient‐clinician trust, time during and outside consultations, an engaged care team, and supplemental resources used outside of the clinic visit
19	Rosenberg et al.^37^		United States	Focus group interview	20 Young breast cancer survivors	Issues affected CPM decision and postsurgical experience	CPM decision	5 Themes: emotions and feelings surrounding surgical decision and surgery, factors affecting the decision, communication and interaction with the healthcare team, impact on postsurgical life and recovery, different support needs
20	Su^38^		China	Semistructured interview	18 Breast cancer patients	Experience of participating in surgical decision making	Surgical decision	6 Themes: patient's perception and attitude towards participation in decision making, process experience of patients involved in surgical decision making, support gained in the process, factors that influence patient decision‐making, psychological reaction of patients involved in surgical decisions, patient needs
21	Peipei et al.^39^		China	Phenomenological method	10 Breast cancer patients	Decision‐making experiences of BR	BR decision	5 Themes: loss of breasts, awakening of self‐consciousness, uncertainty about the information, support from family members, hasty decision making
22	Dicks et al.^40^		Canada	Descriptive qualitative design	35 Breast cancer patients and 13 surgeons	Factors influencing surgical decision	Surgical decision	2 Themes: pushing for mastectomy; factors influencing surgical decisions (clinical factors or genetics, fear or anxiety, family, life stage, cultural influences, experiences of others)
23	Gruß and Mcmullen^9^		United States	Naturalistic observations and semistructured interview	11 Breast cancer patients and 6 clinicians	Barriers to eliciting patient goals and values in SDM	Surgical decision	8 Themes: patients feel well informed and rely on a variety of authoritative sources of information about breast cancer surgery; clinicians emphasize the sharing of biomedical facts; patient contextual factors play an important role in determining how providers share information and elicit patients' goals and values
24	Rosenberg et al.^41^		United States	Semistructured interview	20 ESBC patients	Surgical decision‐making experience	Surgical decision	6 Themes: postsurgical and survivorship concerns; emotional factors; local therapy concerns; reconstruction; recommendations about surgery from providers, family, and friends; family history and genetics
25	Soon et al.^42^		Australia	Semistructured interview	14 Vietnamese‐speaking and 13 English‐speaking patients	Factors that influenced the decision about PMBR	PMBR decision	2 Themes: barriers to undergoing BR (advanced age as a multifaceted barrier, information barriers, concerns and fears regarding surgical procedure, concerns and fears about side‐effects and complications, cancer recurrence); facilitators to undergoing BR (doctor recommendation and attitude, partner influence, able to wear clothing of their choice, psychological facilitators)
26	Yan and Qingyue^43^		China	Semistructured in‐depth interview	16 Patients underwent BR	Experience of receiving BR	BR decision	4 Themes: dream of a wonderful future, decision conflict, demand for social support, decision to be made quickly
27	Lamore et al.^44^		France	Semistructured interview	9 Patients underwent the mastectomy and their partners	BR decision‐making process	BR decision	3 Themes: understanding body modifications, about surgery, medical concerns
28	Min et al.^45^		China	Semistructured in‐depth interview	18 Patients with BCT indications and finally choosing UM	Psychology and influencing factors of decision making for giving up BCT and choosing UM	BCT or UM decision	4 Themes: patient factor, family factor, medical and nursing staff factor, social factor
*Mixed‐method study*								
29	Reaby^46^		Australia	Semistructured interview	95 Breast cancer patients	Mastectomy decision‐making process	Mastectomy decision	3 Themes: decision for mastectomy treatment, alternative surgical options given, consulted others before deciding on mastectomy
30	Durif‐Bruckert et al.^47^		France	Triangulation	205 ESBC patients	Patient perception on SDM about surgical treatment	surgical decision	2 Themes: Avoidance strategies, weight of responsibility
31	Campesino et al.^48^		United States	Quantitative and qualitative study	39 Latina and African‐American breast cancer Survivors	Surgical treatment choices and differences	Surgical decision	6 Themes: clinical indicators, treatment‐related side effects, beliefs regarding recurrence or survival, ascribed role of provider, access to information, lack of health insurance or inability to pay for treatment

Abbreviations: BCT, breast‐conserving therapy; BR, breast reconstruction; CPM, contralateral prophylactic mastectomy; ESBC, early‐stage breast cancer; HCPs, healthcare providers; IBR, immediate breast reconstruction; PMBR, postmastectomy breast reconstruction; RBS, reconstructive breast surgery; RRM, risk‐reducing mastectomy; SDM, shared decision making; UM, unilateral mastectomy.

#### 3.1.2 Study quality

Fourteen of the 31 reviewed studies were rated as high‐quality studies, meeting all the quality criteria (five stars); 16 studies were rated with four stars (Table 3).

**Table 3:** Study quality of the included studies.

ID	Reference	Design	S1	S2	1.1	1.2	1.3	1.4	1.5	5.1	5.2	5.3	5.4	5.5	Score
1	Lally^19^	Qualitative	√	√	√	√	√	√	√						5
2	Begum et al.^20^	Qualitative	√	√	√	√	√	√	√						5
3	Caldon et al.^21^	Qualitative	√	√	?	√	√	√	√						4
4	Fang et al.^22^	Qualitative	√	√	√	√	√	√	√						5
5	Obeidat et al.^23^	Qualitative	√	√	√	√	√	√	√						5
6	Swainston et al.^24^	Qualitative	√	√	√	√	√	√	?						4
7	Abdullah et al.^25^	Qualitative	√	√	√	×	√	√	√						4
8	Obeidat and Lally^26^	Qualitative	√	√	√	√	√	√	√						5
9	Covelli et al.^27^	Qualitative	√	√	√	√	√	√	√						5
10	Potter et al.^28^	Qualitative	√	√	√	√	√	√	√						5
11	Ristevski et al.^29^	Qualitative	√	√	√	√	√	√	√						5
12	Schubart et al.^30^	Qualitative	√	√	√	√	√	√	√						5
13	Robinson et al.^31^	Qualitative	√	√	√	?	√	√	√						4
14	Brown et al.^32^	Qualitative	√	√	√	√	√	√	√						5
15	Fasse et al.^33^	Qualitative	√	√	√	?	√	√	√						4
16	Fu et al.^34^	Qualitative	√	√	√	?	√	√	√						4
17	Gu et al.^35^	Qualitative	√	√	√	√	√	√	√						5
18	Hasak et al.^36^	Qualitative	√	√	√	√	√	√	√						5
19	Rosenberg et al.^37^	Qualitative	√	√	√	√	√	√	?						4
20	Su^38^	Qualitative	√	√	√	√	√	√	√						5
21	Peipei et al.^39^	Qualitative	√	√	√	√	√	√	×						4
22	Dicks et al.^40^	Qualitative	√	√	√	?	√	√	√						4
23	Gruß and Mcmullen^9^	Qualitative	√	√	√	?	√	√	√						4
24	Rosenberg et al.^41^	Qualitative	√	√	√	?	√	?	√						3
25	Soon et al.^42^	Qualitative	√	√	√	?	√	√	√						4
26	Yan and Qingyue^43^	Qualitative	√	√	√	√	√	√	?						4
27	Lamore et al.^44^	Qualitative	√	√	√	√	√	√	√						5
28	Min et al.^45^	Qualitative	√	√	√	?	√	√	√						4
29	Reaby^46^	Mixed	√	√						√	√	√	?	√	4
30	Durif‐Bruckert et al.^47^	Mixed	√	√						√	√	√	?	√	4
31	Campesino et al.^48^	Mixed	√	√						√	×	√	√	√	4

*Note*: √ means yes; × means no; ? means can't tell.

### 3.2 Synthesis of findings

**Figure 2:**
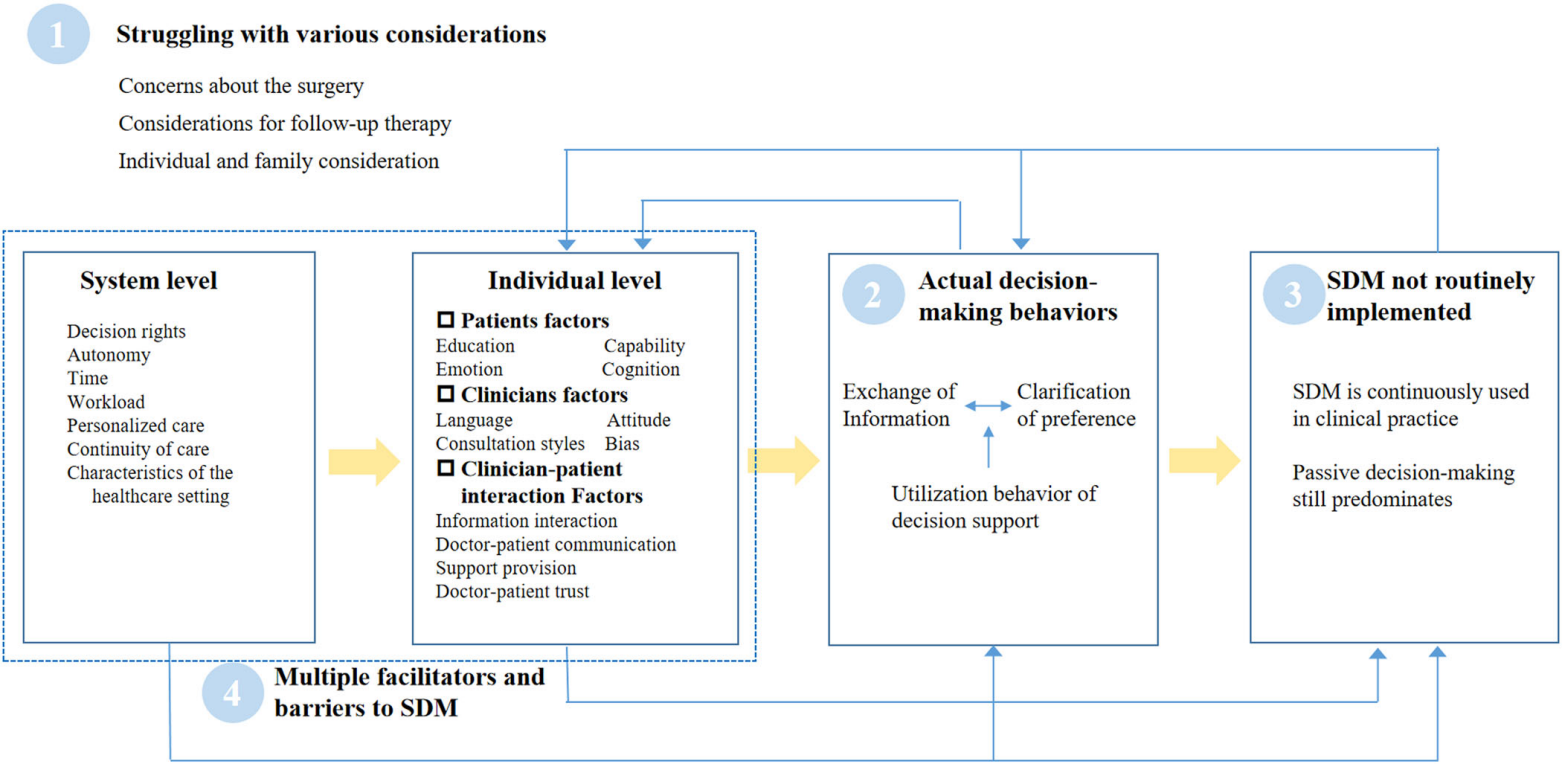
Decision making behaviour, barriers and facilitators. SDM, shared decision making.

Four major themes and 10 subthemes emerged from the selected studies: (a) struggling with various considerations, (b) actual decision‐making behaviours, (c) SDM not routinely implemented and (d) multiple facilitators and barriers to SDM. Table 4 provided details of themes, subthemes, and all codes identified from all included studies. Figure 2 showed how these results map onto the ‘best fit' framework.

**Table 4:** Final themes, subthemes and codes.

Theme	Subthemes	Codes
Theme 1: Struggling with various considerations	Concerns about the surgery	Worrying about cancer recurrence Taking control of cancer Removal of diseased tissue Eliminating risk Survival Prevent recurrence Cosmesis Self‐image Feminine identity BR and prosthesis BR and help start a new life BR and fear of further surgery BR and potential side‐effects
	Considerations for follow‐up therapy	Considerations for adjuvant chemotherapy Considerations for radiotherapy treatment Considerations for hormonal therapy
	Individual and family consideration	Shorter recovery time Intimacy Sexual life Pregnancy Breastfeeding Lack of health insurance Inability to pay
Theme 2: Actual decision‐making behaviours	Information exchange behaviour	Provide the disease details Discuss treatment plan Provide choices Describe pros, cons and potential consequences Information sources Plentiful information Inadequate information Misinformation Too much information
	Clarification of preference	Eliciting personal goals and values Rehearsing the risk and benefits Weighed of different choices Implicit persuasion Hesitant Dilemma
	Utilization behaviour of decision support	Decision‐support components Decision tools (PtDAs) Decision coaching Clinical counselling Insufficient external decision support Adequate external decision support
Theme 3: SDM is not routinely implemented	SDM is continuously used in clinical practice	Patients received detailed information Patients sharing preferences actively Patients deliberated and expressed values actively Clinicians fully considered the patients' values Emphasized decision support
	Passive decision making still predominates	Surgeon made the decision Comply with their surgeon's recommendation Avoid making decisions Misperception of decision making Implicit bias
Theme 4: Multiple facilitators and barriers to SDM	Individual‐level factors	Patient factors Education Capability Emotion Cognition Clinician factors Language Attitude Consultation styles Bias Clinician–patient interaction factors Information interaction Doctor–patient communication Support provision Doctor–patient trust
	System‐level factors	Decision rights Autonomy Time Workload Personalized care Continuity of care Characteristics of the healthcare setting

Abbreviations: BR, breast reconstruction; PtDA, patient decision aids; SDM, shared decision making.

As we can see, several changes were made to the prior framework. ‘Patient with a health condition’ was identified as a crucial step in SDM, rather than a behaviour. Therefore, it was separated into its own component entitled ‘struggling with various considerations’. Additionally, the subthemes of actual decision‐making behaviour were revised based on the coding results. These included three subthemes: (a) information exchange behaviour, (b) clarification of preferences and (c) utilization behaviour of decision support.

#### 3.2.1 Theme 1: Struggling with various considerations

*Concerns about the surgery*: The dominant consideration for patients was worrying about cancer recurrence^21^, ^24^, ^27^, ^30^, ^35^, ^37^, ^38^, ^42^, ^46^ and taking control of cancer.^27^, ^46^ Some women felt that the removal of diseased tissue^21^, ^24^, ^35^, ^37^, ^46^ could relieve their negative emotion, describing it as eliminating risk,^21^, ^27^, ^30^, ^35^ getting rid of it^24^, ^29^, ^35^, ^40^, ^46^ and achieving peace of mind.^23^, ^29^, ^35^, ^37^ Survival was identified as an important issue. Some women thought that UM/MRM would prevent ipsilateral recurrence and CPM would ensure the prevention of contralateral breast cancer.^27^, ^37^, ^48^ Some patients considered their breasts to be important parts of their body image, and losing them after surgery can have many detrimental effects^21^, ^22^, ^27^, ^29^, ^33^, ^35^, ^37^, ^38^, ^39^, ^40^, ^41^ on physical function and psychological function. Regarding BR surgery, some patients expected it to help them start a new life,^43^ while others found it to be a daunting experience.^21^, ^30^, ^34^, ^35^ Some women have expressed fear of undergoing further surgery^34^, ^42^ and the potential risks of the implants used in reconstruction.^34^, ^42^

*Considerations for follow‐up therapy*: Some patients considered the impacts of follow‐up therapy on their lives, including the risks and side effects of adjuvant chemotherapy, RT, and hormonal therapy.^24^, ^27^, ^30^, ^35^, ^38^, ^44^, ^48^ Some held firm opinions that choosing BCT would result in struggling with radiation therapy and unwanted side effects, such as nausea and vomiting.

*Individual and family considerations*: Breasts played an important role in marriage and childrearing as a ‘wife’ and ‘mother’.^34^, ^37^, ^41^, ^43^ Therefore, women had many considerations about the loss of the breast. Intimacy, sexual life, pregnancy, and breastfeeding were some important issues^37^, ^41^, ^44^ that patients and their families focused on. Some patients tended to choose the least disruptive choice,^19^, ^21^, ^35^, ^38^, ^41^, ^42^ such as choosing a treatment plan with a shorter recovery time or less overall negative effect on their life. Some patients expressed concern that a prolonged recovery period would impact their ability to care for their young children^40^, ^44^ and return to work.^37^, ^38^, ^41^, ^42^, ^44^ Additionally, for some patients, lack of health insurance or inability to pay for some treatment expenses also affected the choice of therapy.^39^, ^43^, ^45^, ^48^

#### 3.2.2 Theme 2: Actual decision‐making behaviours

*Information exchange behaviour*: Information was a prerequisite and the exchange of information played a significant role. Patients regarded HCPs as their primary source of information. Clinician was frequently mentioned as trusted source,^21^, ^24^, ^25^, ^27^, ^28^, ^30^, ^37^, ^38^, ^39^, ^46^ while BCN and CNS^9^, ^26^, ^28^, ^48^ reiterated and reinforced patients' information needs.^21^ They often used some sort of diagrams or pictures to explain the therapy.^9^, ^24^, ^30^ Some women reported feeling a lack of choice in their treatment options^20^, ^36^ as their surgeons only provided recommendation therapy.^29^ These women felt they were not adequately informed about the choice and were unprepared to make decisions.^20^, ^21^, ^37^, ^42^, ^48^ Other sources of information included family,^19^, ^23^, ^25^, ^27^, ^30^, ^34^, ^35^, ^37^, ^38^, ^41^, ^46^ friends,^19^, ^23^, ^25^, ^27^, ^30^, ^34^, ^37^, ^41^, ^46^ colleagues,^30^, ^37^, ^41^ breast cancer survivors,^26^, ^30^, ^37^, ^41^ books,^19^, ^23^, ^24^, ^25^, ^26^, ^37^, ^48^ brochures,^23^, ^26^ and internet.^9^, ^19^, ^23^, ^24^, ^25^, ^26^, ^28^, ^30^, ^34^, ^37^, ^45^, ^48^ However, some women reported a myriad of inaccurate or noncredible information.^23^, ^34^, ^39^, ^45^ Additionally, some patients found that an excess of information could be challenging to fully comprehend.^30^, ^39^

*Clarification of preference*: The significance of eliciting personal preferences^23^ and values^9^ for SDM has been widely acknowledged. The deliberation process involved rehearsing the risks and benefits and identifying reasons in favour of the decision.^32^, ^37^ Clinicians typically spend a certain amount of time discussing^21^ and consulting^20^, ^27^ with patients and their families. It also put a higher demand on the doctor's work. It is important to note that certain scenarios, such as implicit persuasion, may cause decision biases in this process.^36^ For instance, clinicians stressing or minimizing certain cancer characteristics may influence patients' decision making.^21^ Some women may be hesitant to express their preference and feel unsure about their decision.^22^, ^44^ Although some patients found the decision‐making process challenging, they often felt proud of their achievement upon reflection.^21^ Failure to fully understand information about risks and benefits, or focusing only on their positive or negative aspects, can lead to difficulties in decision making.^20^

*Utilization behaviour of decision support*: The decision support included several components, such as family support,^19^, ^22^, ^23^, ^24^, ^30^, ^37^, ^38^, ^39^, ^43^, ^46^ peer support,^37^, ^38^, ^43^ psychological support,^21^, ^23^, ^25^, ^30^, ^33^, ^37^ information support,^19^, ^21^, ^24^, ^27^, ^28^, ^30^, ^33^, ^34^, ^37^, ^39^, ^46^ and professional support.^32^, ^43^ Decision support^49^ refers to providing decision tools, decision coaching, and clinical counselling to patients to enhance their ability to cope with medical treatment. Clinical counselling played a crucial role in resolving treatment problems and contradictions faced by patients.^32^, ^43^ However, the brief information provided by clinicians during limited counselling time may not meet patients' information needs. Patient decision aids (PtDAs) were often accessible through online resources, information packets, and manuals. Some women received take‐home PtDAs^24^ and found them adequate and helpful. However, some patients reported they had difficulty in finding adequate external decision support resources.^39^ In clinical practice, decision coaches were typically nurses, social workers, and health educators, although this approach was less commonly used. Overall, many patients felt that the decision support provided to them was insufficient.^37^, ^39^

#### 3.2.3 Theme 3: SDM not routinely implemented

*SDM is continuously used in clinical practice*: Some patients described SDM as a process of sharing personal experiences and preferences.^36^, ^38^ On the premise that patients deliberated and expressed their values, clinicians fully considered the patients' values and emphasized decision support, which can contribute to the realization of SDM.^21^, ^25^ Some women received detailed information during the consultation, as well as take‐home decision aids.^24^ After introducing the concept and rationale of using SDM early in the consultation, the implementation of SDM was smoother during the doctor–patient interaction process.^21^, ^24^, ^36^ In general, women who are highly educated, proactive in seeking information, fluent in English, have healthcare knowledge and actively connect with HCPs are empowered to be active decision makers.^23^, ^38^

*Passive decision making still predominates*: Some women pointed out that it was their surgeon who made the decision, and their primary responsibility was to understand and comply with their surgeon's recommendation.^39^, ^46^ Some felt that they lacked choice, even when making preference‐sensitive decisions.^20^, ^36^ Some patients exhibited a set of avoidance strategies that enabled them to avoid making decisions.^20^, ^23^, ^24^, ^26^, ^46^, ^47^ They adopted passive decision‐making methods due to some misperception.^24^, ^46^ Some women thought that ‘left the decision to the clinician' meant trusting the clinician. Also, it would not compromise the clinician's expertise and authority and also avoid self‐anxiety and worry.^24^, ^47^ They felt that they were not qualified to ask questions about medical issues because of the lack of professional medical knowledge.^26^ The shock of diagnosis and making decisions in a short time were described by some women as reasons for not engaging further.^24^ Generally speaking, older women, or those less educated, and less fluent in English, recently immigrated complied more readily with their surgeon's recommendation.^23^, ^26^

#### 3.2.4 Theme 4: Multiple facilitators and barriers to SDM

Barriers and facilitators influencing SDM included individual‐level factors (patient factors, clinician factors and clinician–patient interaction factors) and system‐level factors.

##### Individual‐level factors

*Patient factors*: In general, well‐educated women were more motivated to seek out access to additional support sources, and this appeared to positively influence their SDM experience.^28^ Patients' decision‐making ability was a prerequisite for participation. Language barriers^21^, ^42^, ^48^ and having difficulty grasping formal medical information^28^, ^47^ were barriers for SDM. And decision‐making behaviours were affected by the emotional response^20^, ^22^, ^23^, ^24^, ^28^, ^30^, ^32^, ^33^, ^38^, ^39^, ^40^, ^41^, ^45^, ^46^, ^47^ like shock and fear. There were misconceptions in the decision‐making process. Passive decision making role often seemed from various misperceptions^20^, ^23^, ^24^, ^26^, ^36^, ^38^, ^39^, ^46^, ^47^ such as ‘left the decision to the doctor’ and ‘patients were not qualified to ask the question’. It was worth mentioning that some patients had realized the importance of self‐participation in the decision making, and they actively communicated with doctors and sought advice from survivors to broaden their information sources.^38^

*Clinician factors*: Clinicians' consultation styles varied from open, tailored, two‐way dialogues to a more prescriptive style.^21^ Language varied from everyday to bio‐medical. The language and consultation styles adopted by clinicians influenced the accessibility of information to patients.^21^ The surgeon's attitude, which was friendly, approachable, and comfortable, can establish trust and great communication effect.^29^, ^32^ Some patients felt that clinicians were biased^36^ in their presentation of options because of the patient's age, race, or socioeconomic status.

*Clinician–patient interaction factors*: Positively/negatively clinician–patient interaction quality^21^, ^37^ affected the practice of SDM. Misinformation,^23^ insufficient support resources^20^, ^34^, ^36^, ^38^, ^42^, ^47^, ^48^ and lack of communication^21^, ^26^, ^37^, ^38^, ^45^ were barriers for SDM. Also, there was cognitive bias^45^ between preoperative information provided by HCPs and comprehended by patients. Also, the quality and quantity of information greatly impacted the patients' behaviour. Access to an abundance of information and supplemental resources was a strong motivating factor for being involved in SDM.^34^, ^36^, ^43^ Some patients expressed trust in HCPs' recommendations^19^, ^20^, ^23^, ^29^, ^36^, ^37^, ^42^, ^46^ and appreciated the effective and helpful communication.^20^, ^24^, ^36^, ^37^, ^42^, ^46^ However, sometimes this trust was described by some women as enabling them to take a passive role.^24^

##### System‐level factors

Patients' autonomy and decision rights were primary environmental considerations about implementing SDM. Decision rights^21^ were closely related to characteristics of the healthcare setting. At the same time, sufficient time for communication between clinicians and patients could promote the implementation of SDM. However, the lack of time^21^ and rushed decision making^28^, ^38^, ^39^, ^43^, ^46^ due to the workload were detrimental to the overall quality of medical care.^37^ It was challenging to accomplish all of this within such a limited surgical consultation time.^9^ SDM was an effective technique to provide patient‐centred care.^36^ The commonness of diseases and the individuality of patients suggested that HCPs needed to understand the patients' preference.^33^ Personalized care and continuity of care could better help patients to explain their values^33^ and be involved in their disease management.^30^, ^47^

## 4 DISCUSSION

Findings from this review reveal four main themes describing the patient's decision‐making behaviour and SDM‐related barriers and facilitators. The results of this study suggest that although SDM has occupied a certain proportion, paternalistic decision‐making style is still the main decision‐making style. There are many barriers and facilitators to the implementation of SDM, such as individual‐level and system‐level factors. This study helps to discover and overcome unwarranted variations such as information bias and implicit bias, to empower patients to consider treatment choices and integrate patient values and medical evidence to facilitate SDM and improve the quality of care in the clinical practice.

This study finds that paternalistic decision making still occupies a certain proportion, especially among older women or those less educated. Previous systematic review^11^ also pointed out that SDM in surgery was still in its infancy, and the use of SDM within the surgical practice was infrequent. Some studies^12^, ^50^ found that there were some improper cognitions in the surgical decision‐making process, which was similar to our study. It was revealed that some patients avoided participating in treatment decisions on the grounds of doctors' authority and trusting clinicians,^24^, ^47^ which was contrary to the idea of SDM.^51^ As can be seen, these are implicit biases^52^ related to medical decision making. According to dual‐process theory^53^ and Tyler's study,^54^ when individuals face time constraints and uncertainty, their decision making may be influenced by heuristics or cognitive shortcuts. Heuristics may lead to bias or cognitive errors. Under time or other pressures, people are more likely to rely on intuition for decision making. Given the opportunity to deliberate, people^53^ are more likely to rely on a rational comparison of risks and benefits. Therefore, SDM still needs to be further promoted^11^ in clinical practice.

The complexity of surgical decision making requires patients to consider multiple components and necessitates adequate information exchange. However, our study found that healthcare professionals provided limited information, and external sources often provided inaccurate or non‐credible information. The findings are concerning because inadequate or low‐quality information can result in ineffective decision making. Furthermore, our study revealed the existence of implicit persuasion during the preference clarification process, which aligns with previous research.^55^ Additionally, Whyte et al.'s^12^ study identified multiple cognitive and behavioural biases, including herding bias and confirmation bias, present in a large portion of the sample of breast cancer patients, nurses and surgeons. This may occur because surgeons sometimes do not have time^9^ to discuss these complex trade‐offs with patients. Therefore, empowering patients and improving the efficiency of the discussion^9^ are important steps to overcome such challenges. This study finds that patients have problems during this process such as limited support approach, insufficient support materials, lack of professional decision support, and so forth. Decision support is both a challenge and an opportunity in the field of healthcare. Therefore, it is particularly important to develop PtDAs^7^ and train decision coaches^56^ to identify vulnerable groups with deficits in surgical decision making, and improve patients' decision preparation.

This study suggests that there are some barriers and facilitators in the process of shared surgical decision making, including micro (individual) level factors. Our study reports that patients' individual factors such as decision‐making ability, emotion, educational level, and decision‐making cognition will affect the implementation of SDM. The theory of Planned Behaviour^57^ also suggested that behaviours were not only affected by behavioural intention but also restricted by actual control conditions such as personal ability. Significantly, most patient‐related factors are potentially modifiable, and many could be addressed by attitudinal changes at the levels of the patient. Our review also finds some clinicians‐related factors such as consultation style and clinician's attitude. There is a general consensus that clinicians‐related factors^5^ remain the most often cited barrier and facilitators for implementing SDM in clinical practice across many different cultural and organizational contexts. In some cases, this sharing of the decision‐making process is not only influenced by the doctor and the patient factor but also clinician–patient interaction factors.^5^ Covvey et al.'s^50^ study also found that in the oncology treatment context, major barriers from the patient perspective included poor clinician–patient communication. Major facilitators of SDM included physician consideration of patient preferences, positive physician actions/behaviours, and the use of support systems. Annabel's study^5^ also demonstrated that the patient–doctor relationship, particularly trust, was identified as a significant factor. This is similar to the results of our study. Trust in the physician or health system has been identified as a key factor not just in patient satisfaction with their care but also in their decision to accept or adhere to treatment at all.^58^

In this study, it is found that meso‐ and macrolevel factors such as time, workload, decision‐making power, continuity of care and characteristics of the healthcare setting are the main influencing factors for the clinical implementation of SDM. Waddell et al.'s^59^ research in the hospital context also found that organization‐ and system‐related factors also included the culture of the organization and leaders engaging in SDM. Also, changing clinical guidelines to promote SDM was reported by clinicians and other stakeholders as being one way in which the system could be changed to facilitate SDM.^59^ Scholl et al.'s^58^ scoping review also found that factors associated with the success of SDM implementation include adequate resourcing, setting of SDM as a priority, integration of SDM into teams and workflow, and cultural and organizational leadership, whereas the system‐level factors include clinical guidelines, incentives, education, licensing, culture and policy. This is not reported in this study, which may be due to the small situation focused in this study.

### 4.1 Study limitations

This study had several limitations. First, although eight databases were searched, it is possible that some eligible studies were missed. Second, only articles published in English and Chinese were included, and the experiences of participants from other language backgrounds may not be well analyzed. This may have limited the number of articles identified. Third, due to the extensive literature in this research field, we only included qualitative evidence for data synthesis.

### 4.2 Clinical implications

For high‐value procedures, patients should demonstrate a full understanding of risks, benefits and alternatives, and there should be concordance between patient preferences and expected behaviours. The study indicates a discrepancy between information exchange behaviours, value clarification, decision support utilization, and SDM due to cognitive and behavioural biases. It is important to avoid cognitive biases that may affect medical decision making. In the future, surgeons should actively interact with their patients and patients should proactively express their personal values and priorities. Additionally, this study provides insight into the barriers and facilitators of SDM in breast cancer surgical treatment. The information gained from this study may be useful in expanding on possible interventions and implications for the global public. Being able to weigh up both barriers and facilitators would aid practical steps in improving SDM for breast cancer surgery.

## 5 CONCLUSION

Surgical decision making was an important step in the patient's medical treatment process, which affected the patient's health outcome and experience. Patients' surgical decision‐making styles were various, while paternalistic decision‐making style has still occupied a certain proportion. The surgical decision‐making process was a complex cycle process, with the interweaving and interaction of cognitions and behaviours. When some individuals made decisions, their behaviours were affected by individual‐level and system‐level factors. Identify the current state of decision‐making behaviours and provide targeted professional decision‐support tools that can help facilitate the implementation of SDM in clinical practice. Therefore, HCPs and other stakeholders should constantly improve their communication skills and emphasize the importance of decision support to promote the implementation of SDM.

## AUTHOR CONTRIBUTIONS

**Hongying Zheng**: Conceptualization; methodology; software; data curation; formal analysis; visualization; writing—review and editing; writing—original draft. **Linning Yang**: Methodology; data curation; software; formal analysis. **Jiale Hu**: Conceptualization; methodology; formal analysis; supervision; writing—review and editing. **Yan Yang**: Conceptualization; methodology; formal analysis; supervision; writing—review and editing.

## CONFLICT OF INTEREST STATEMENT

The authors declare no conflict of interest.

## ETHICS STATEMENT

This is a systematic review. Ethics approved is not applicable.

## Supporting information

Supporting information.

## Data Availability

The data that support the findings of this study are available from the corresponding author upon reasonable request.
