# Supplementary material for: Behaviour, barriers and facilitators of shared decision making in breast cancer surgical treatment: A qualitative systematic review using a ‘Best Fit’ framework approach
Source: Health Expect. 2024 Apr 1;27(2):e14019. doi: 10.1111/hex.14019 (PMC10982676; doi:10.1111/hex.14019)
Supplement: Supplementary file 1 — Supporting information. [file HEX-27-e14019-s001.doc]

**Supplemental Material**

**Search strategy**

Pubmed medline

| Pubmed medline | | |
| --- | --- | --- |
| #1 | shared decision*[tiab] or sharing decision*[tiab] or informed decision*[tiab] or informed choice*[tiab]or decision aid*[tiab] or ((share*[ti] or sharing*[ti] or informed*[ti]) and (decision*[ti] or deciding*[ti] or choice*[ti])) |  |
| #2 | decision making[mh:noexp] or decision support techniques[mh:noexp] or decision support systems, clinical[mh] or choice behaviour[mh:noexp] or decision making*[tiab] or decision support*[tiab] or choice behaviour*[tiab] or ((decision*[ti] or choice*[ti]) and (making*[ti] or support*[ti] or behaviour*[ti])) |  |
| #3 | patient participation[mh] or patient participation*[tiab] or consumer participation*[tiab] or patient involvement*[tiab] or consumer involvement*[tiab] or ((patient*[ti] or consumer*[ti]) and (involvement*[ti] or involving*[ti] or participation*[ti] or participating*[ti])) |  |
| #4 | Breast Neoplasms[mh] or breast cancer[tiab] or breast neoplasm[tiab] or breast carcinoma[tiab] or breast malignancy[tiab] or breast tumor*[tiab] or breast tumour*[tiab] |  |
| #5 | surg*[tiab] or operat*[tiab] |  |
| #6 | (#1 OR (#2 AND #3)) AND #4 AND #5 | 473 |

Embase

| Embase | | |
| --- | --- | --- |
| #1 | 'shared decision':ti,ab OR 'sharing decision':ti,ab OR 'informed decision':ti,ab OR 'informed choice':ti,ab OR 'decision aid':ti,ab OR ((share*:ti OR sharing*:ti OR informed*:ti) AND (decision*:ti OR deciding*:ti OR choice*:ti)) |  |
| #2 | 'clinical decision making'/exp OR 'decision making'/exp OR 'decision support system'/exp OR 'ethical decision making'/exp OR 'family decision making'/exp OR 'medical decision making'/exp OR 'patient decision making'/exp OR 'decision making':ti,ab OR 'decision support':ti,ab OR 'choice behaviour':ti,ab OR ((decision*:ti OR choice*:ti) AND (making*:ti OR support*:ti OR behaviour*:ti)) |  |
| #3 | 'patient participation'/exp OR 'patient participation':ti,ab OR 'consumer participation':ti,ab OR 'patient involvement':ti,ab OR 'consumer involvement':ti,ab OR ((patient*:ti OR consumer*:ti) AND (involvement*:ti OR involving*:ti OR participation*:ti OR participating*:ti)) |  |
| #4 | 'breast cancer':ab,ti OR 'breast neoplasm':ab,ti OR 'breast carcinoma':ab,ti OR 'breast malignancy':ab,ti OR 'breast tumor':ab,ti |  |
| #5 | 'surg*':ab,ti OR 'operat*':ab,ti |  |
| #6 | (#1 OR (#2 AND #3)) AND #4 AND #5 | 501 |

CINAHL

| CINAHL | | |
| --- | --- | --- |
| #1 | AB Shared Decision* OR TI Shared Decision*OR AB Sharing Decision* OR TI Sharing Decision* OR AB Informed Decision* OR TI Informed Decision* OR AB Informed Choice* OR TI Informed Choice* OR AB Decision Aid* OR TI Decision Aid* OR ((TI Share* OR TI Sharing OR TI Informed*) AND (TI Decision* OR TI Deciding* OR TI Choice*)) |  |
| #2 | MH “Decision Making+” OR MW Decision Support OR AB Decision Making* OR TI Decision Making* OR AB Decision Support* OR TI Decision Support* OR AB Choice Behaviour* OR TI Choice Behaviour* OR ((TI Decision* OR TI Choice*) AND (TI Making* OR TI Support* OR TI Behaviour*)) |  |
| #3 | MH Consumer Participation OR AB Patient Participation* OR TI Patient Participation* OR AB Consumer Participation* OR TI Consumer Participation* OR AB Patient Involvement* OR TI Patient Involvement* OR AB Consumer Involvement* OR TI Consumer Involvement* OR ((TI Patient* OR TI Consumer*) AND (TI Participating* OR TI Participation* OR TI Involving* OR TI Involvement*)) |  |
| #4 | TI breast cancer OR TI breast neoplasms OR TI breast carcinoma OR TI breast malignancy OR TI breast tumor OR TI breast tumour |  |
| #5 | AB surg* OR AB operat* |  |
| #6 | (S1 OR (S2 AND S3)) AND S4 AND S5 | 168 |

Cochrane Library

| Cochrane Library | | |
| --- | --- | --- |
| #1 | "Shared Decision*" or "Sharing Decision*" or "Informed Decision*" or "Informed Choice*" or "Decision Aid*" or ((Share*OR Sharing* or Informed*) and (Decision* or Deciding* or Choice*)):ti,ab,kw |  |
| #2 | "Decision Making*" or "Decision Support*" or "Choice Behaviour" or ((Decision* or Choice*) and (Making* or Support* or Behaviour*)):ti,ab,kw |  |
| #3 | "Patient Participation*" or "Consumer Participation*" or "Patient Involvement*" or "Consumer Involvement*" or ((Patient* or Consumer*) and (Involvement* or Involving* or Participation* or Participating*)):ti,ab,kw |  |
| #4 | (breast cancer or breast neoplasm* or breast carcinoma or breast malignancy or breast tumor* or breast tumour*):ti,ab,kw |  |
| #5 | (surg* or operat*):ti,ab,kw |  |
|  | Limit to trials |  |
| #6 | (#1 or (#2 and #3)) and #4 and #5 | 241 |

Wangfang

| Wangfang | | |
| --- | --- | --- |
| #1 | 主题：（“共享决策”OR“共同决策”OR“决策辅助”OR“决策偏好”OR“决策支持”OR“决策参与”） |  |
| #2 | 主题：（“乳腺癌”OR“乳腺肿瘤”） |  |
| #3 | 主题：（“手术”OR“外科”） |  |
| #4 | 限定文献类型期刊论文、学位论文、会议论文 |  |
| #5 | #1 and #2 and #3 and #4 | 15 |

CNKI

| CNKI | | |
| --- | --- | --- |
| #1 | 主题：（共享决策 + 共同决策 + 决策辅助 + 决策偏好 + 决策支持 + 决策参与） |  |
| #2 | 主题：（乳腺癌 + 乳腺肿瘤） |  |
| #3 | 主题：（手术 + 外科） |  |
| #4 | #1 and #2 and #3 | 11 |

CBM 2021.3.22

| CBM | | |
| --- | --- | --- |
| #1 | ("手术"[摘要:智能] OR "外科"[摘要:智能]) AND ("乳腺癌"[摘要:智能] OR "乳腺肿瘤"[摘要:智能]) AND ("共享决策"[摘要:智能] OR "共同决策"[摘要:智能] OR "决策辅助"[摘要:智能] OR "决策偏好"[摘要:智能] OR "决策支持"[摘要:智能] OR "决策参与"[摘要:智能]) | 8 |
